# Metabolomics Profiling for Obstructive Sleep Apnea and Simple Snorers

**DOI:** 10.1038/srep30958

**Published:** 2016-08-02

**Authors:** Huajun Xu, Xiaojiao Zheng, Yingjun Qian, Jian Guan, Hongliang Yi, Jianyin Zou, Yuyu Wang, Lili Meng, Aihua Zhao, Shankai Yin, Wei Jia

**Affiliations:** 1 Department of Otolaryngology Head and Neck Surgery & Center of Sleep Medicine, Shanghai Jiao Tong University Affiliated Sixth People’s Hospital, Yishan Road 600, 200233 Shanghai, China; 2 Otolaryngological Institute of Shanghai Jiao Tong University, Yishan Road 600, 200233 Shanghai, China; 3 Clinical Research Center, Shanghai Jiao Tong University School of Medicine, South Chongqing Road 225, 200020 Shanghai, China; 4 Center for Translational Medicine, Shanghai Jiao Tong University Affiliated Sixth People’s Hospital, Yishan Road 600, 200233 Shanghai, China

## Abstract

Few clinical studies have explored altered urinary metabolite levels in patients with obstructive sleep apnea (OSA). Thus, we applied a metabolomics approach to analyze urinary metabolites in three groups of participants: patients with polysomnography (PSG)-confirmed OSA, simple snorers (SS), and normal subjects. Ultra-performance liquid chromatography coupled with quadrupole time-of-flight mass spectrometry and gas chromatography coupled with time-of-flight mass spectrometry were used. A total of 21 and 31 metabolites were differentially expressed in the SS and OSA groups, respectively. Patients with OSA had 18 metabolites different from those with SS. Of the 56 metabolites detected among the 3 groups, 24 were consistently higher or lower. A receiver operator curve analysis revealed that the combination of 4-hydroxypentenoic acid, arabinose, glycochenodeoxycholate-3-sulfate, isoleucine, serine, and xanthine produced a moderate diagnostic score with a sensitivity (specificity) of 75% (78%) for distinguishing OSA from those without OSA. The combination of 4-hydroxypentenoic acid, 5-dihydrotestosterone sulfate, serine, spermine, and xanthine distinguished OSA from SS with a sensitivity of 85% and specificity of 80%. Multiple metabolites and metabolic pathways associated with SS and OSA were identified using the metabolomics approach, and the altered metabolite signatures could potentially serve as an alternative diagnostic method to PSG.

Obstructive sleep apnea (OSA) is characterized by a history of habitual snoring and repeated nocturnal upper airway obstruction and is a highly prevalent sleep disorder (i.e., 2% of men and 4% of women)^1^,^2^. Notable sequelae of OSA are cardiovascular and metabolic consequences, including disturbed lipid metabolism and insulin resistance^3^,^4^,^5^. A history of snoring is believed to be an early sign of OSA, affecting about 30% of the general population^6^. Although complaining of snoring is the most common clinical manifestation of patients with OSA, OSA but not snoring is associated with a high incidence of cardiovascular events and all-cause mortality^7^,^8^. Many studies have attempted to identify the pathogenesis of OSA through multiple pathways, including inflammation and oxidative stress, using altered levels of various biomarkers in biofluids. Understanding the metabolic signature shift in patients with OSA is important to develop preventive strategies and therapeutic interventions. Fatty acids, carbohydrates, and amino acids are common metabolites involved in cellular physiology, structure, signaling, and survival. Unfortunately, traditional technologies have detected a paucity of specific biomarkers. Thus, new technologies should be developed to offer greater insight into the understanding of the biochemical mechanisms of early-stage OSA.

Metabolomics is a high-sensitivity, high-throughput profiling method with which to study the characteristic changes in low-molecular-weight metabolites in a pathophysiological state. The primary aim of such an approach is to explore novel biomarkers and identify physiological and pathological mechanistic processes. Metabolomics has been increasingly applied to many pulmonary and sleep disorders^9^,^10^,^11^,^12^. The metabolomics analytical platform often includes nuclear magnetic resonance spectroscopy as well as mass spectrometry coupled with gas chromatography or liquid chromatography.

To date, a limited number of small-sample-size metabolomics studies have been performed to explore metabolomics profiling and the underlying mechanisms in OSA^13^,^14^,^15^. Thus, we utilized a combination of ultra-performance liquid chromatography coupled with quadrupole time-of-flight mass spectrometry (UPLC-Q-TOF-MS) and gas chromatography coupled with time-of-flight mass spectrometry (GC-TOF-MS) to investigate: the metabolic changes occurring during the development of OSA, the mechanistic pathways involved in OSA, and candidate metabolite markers useful for diagnosing OSA.

## Results

### Basic characteristics

**Figure 1:**
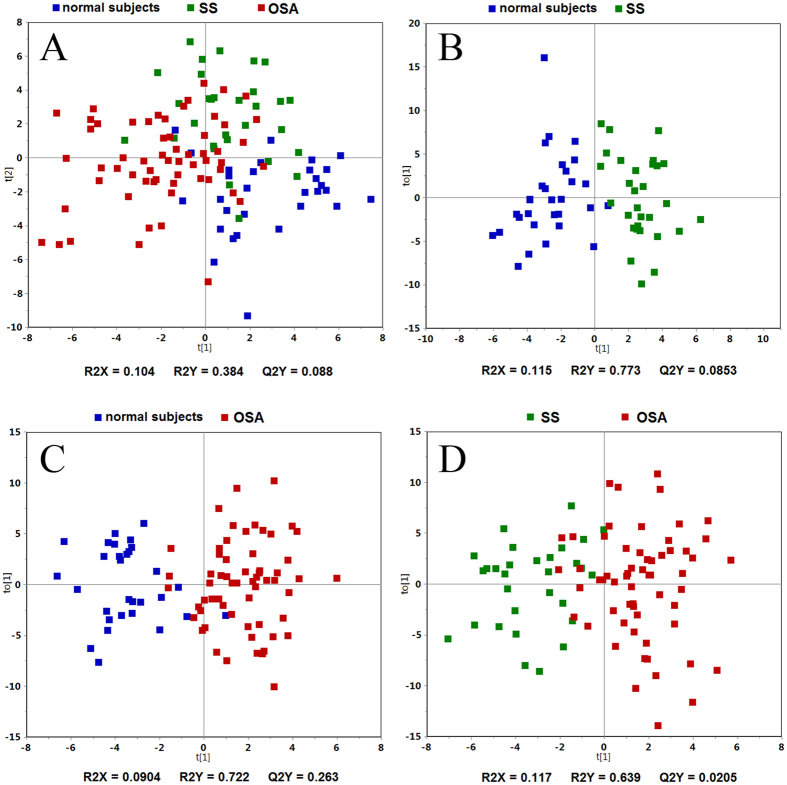
Score plots of the orthogonal partial least-squares discriminant analysis model for the obstructive sleep apnea (OSA), simple snorers (SS), and control groups. (**A**) The model parameters were: R^2^*X* = 0.104, R^2^*Y* = 0.384, and Q^2^*Y* = 0.088; (**B**) the model parameters were: R^2^*X* = 0.115, R^2^*Y* = 0.773, and Q^2^*Y* = 0.085; (**C**) the model parameters were: R^2^*X* = 0.090, R^2^*Y* = 0.722, and Q^2^*Y* = 0.263; (**D**) the model parameters were: R^2^*X* = 0.117, R^2^*Y* = 0.639, and Q^2^*Y* = 0.021.

In total, 120 subjects (60 with OSA, 30 were simple snorers (SS), and 30 normal subjects) were included in the metabolomics analyses. As presented in Table 1, no differences in age, sex, or body mass index (BMI) were observed among the three groups. Patients with SS and OSA had poorer performance during the polysomnography (PSG) and the biochemical indices were also more severe in patients with OSA than those in the control group. The demographic, anthropometric, and PSG findings are presented in Table 1. The orthogonal partial least-squares discriminant analysis (OPLS-DA) model demonstrated clear separation between OSA, SS, and normal subjects (Fig. 1, parameters for the OPLS-DA model).

### Metabolic profiles associated with OSA and SS from normal subjects

We identified 21 metabolites that distinguished the SS group from normal subjects. (Supplementary Table 1, variable importance in projection (VIP) > 1 and *p* < 0.05, respectively). Of them, most of the fatty acids and metabolomics profiling of phospholipid biosynthesis increased significantly. Levels of aspartyl-serine, isoleucine-threonine (Ile-Thr), and methionine increased, whereas those of 3-hydroxyanthranilic acid and 5-hydroxytryptophan level decreased. Other carbohydrate metabolism intermediates in the tricarboxylic acid cycle (TCA), indoles and derivatives, glutamate metabolites, steroids, and nucleic acid metabolite were also altered significantly.

In total, 31 metabolites differentiated the OSA group from the normal subjects. The altered metabolites are listed in Supplementary Table 2 (VIP > 1 and *p* < 0.05, respectively). Similarly, fatty acids levels increased significantly, including 2,4-dihydroxybutyric acid, 2-hydroxy-3-methylbutyric acid, 3,4-dihydrxoybutyric acid, 6-aminocaproic acid, pentanoic acid, and glyceraldehyde, whereas the bile acid glycochenodeoxycholate-3-sulfate (GCDCA-3-sulfate) decreased. Five amino acids increased significantly, and two (methylcysteine and serine) decreased. Moreover, phospholipid biosynthesis, carbohydrate metabolism, TCA, glutamate metabolism, nucleic acid metabolism, indoles and derivatives, and spermine and tyrosine metabolism were also altered significantly.

In summary, 18 metabolites differentiated patients with OSA from those with SS. Additionally, most fatty acids (i.e., 3-hydroxybutyric acid, 3-methyl-3-hydroxybutyric acid, and 4-hydroxypentenoic acid) and TCA increased, whereas most amino acid and nucleic acid decreased. The metabolomics profiling of steroidogenesis, spermine biosynthesis, tryptophan metabolism, and porphyrin metabolism were altered significantly (Supplementary Table 3, VIP > 1 and *p* < 0.05, respectively).

### Metabolic shift from normal subjects to OSA

**Figure 2:**
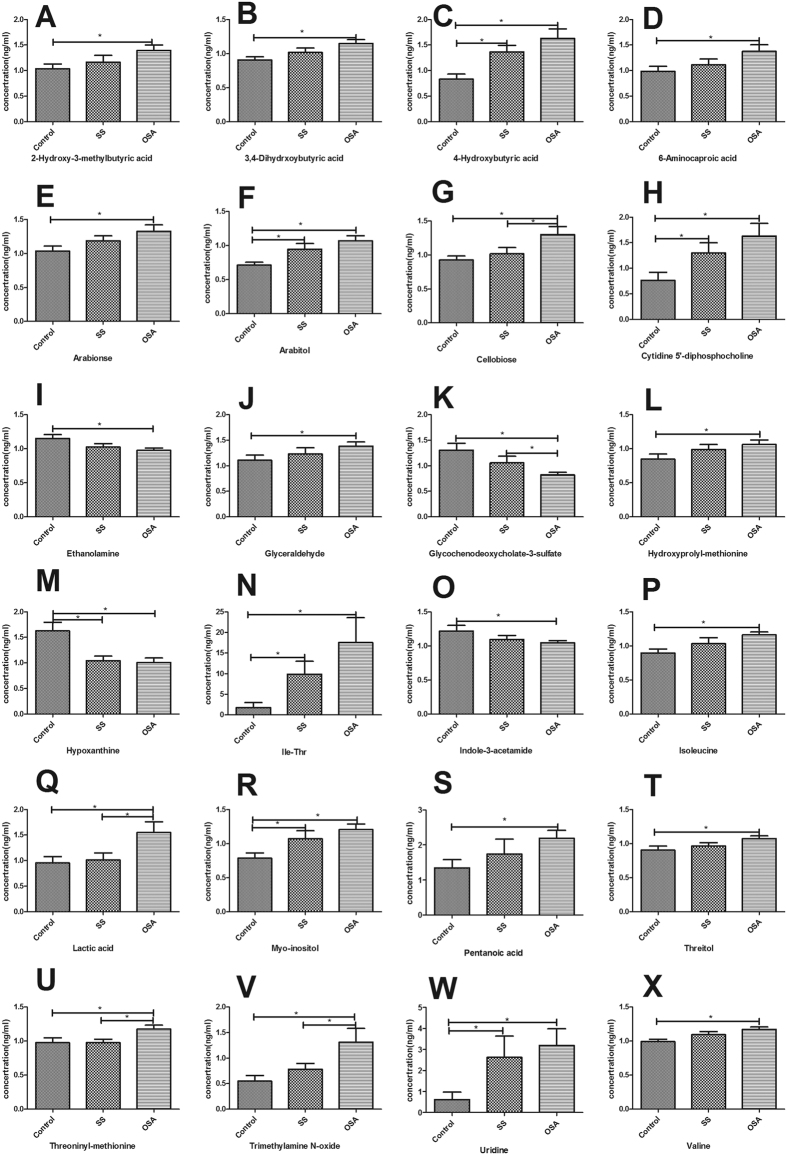
24 metabolites (2-hydroxy-3-methylbutyric acid, 3,4-dihydrxoybutyric acid, 4-hydroxybutyric acid, 6-aminocaproic acid, arabionse, arabitol, cellobiose, cytidine 5′-diphosphocholine, ethanolamine, glyceraldehyde, glycochenodeoxycholate-3-sulfate, hydroxyprolyl-methionine, hypoxanthine, Ile-Thr, indole-3-acetamide, isoleucine, lactic acid, myo-inositol, pentanoic acid, threitol, threoninyl-methionine, trimethylamine N-oxide, uridine, valine) were consistently higher or lower.

Of the differentially produced metabolites among the 3 groups, 24 metabolites (2-hydroxy-3-methylbutyric acid, 3,4-dihydrxoybutyric acid, 4-hydroxybutyric acid, 6-aminocaproic acid, arabionse, arabitol, cellobiose, cytidine 5′-diphosphocholine, ethanolamine, glyceraldehyde, GCDCA-3-sulfate, hydroxyprolyl-methionine, hypoxanthine, Ile-Thr, indole-3-acetamide, isoleucine, lactic acid, myo-inositol, pentanoic acid, threitol, threoninyl-methionine, trimethylamine N-oxide (TMAO), uridine, and valine) were consistently higher or lower (Fig. 2A–X).

### Potential OSA diagnostic panel

**Figure 3:**
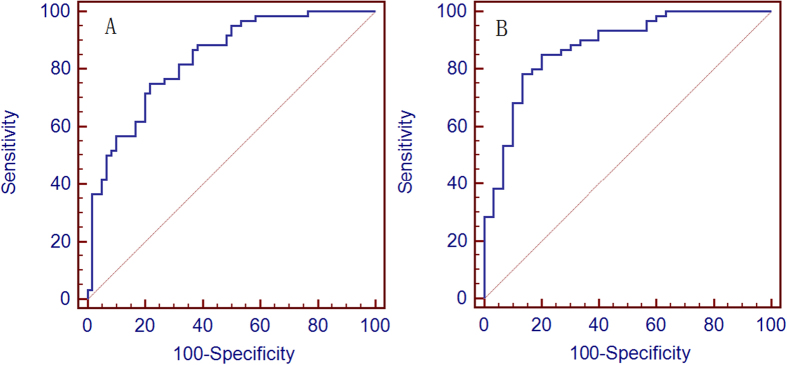
Receiver-operator characteristic (ROC) curve of the composite biomarker panel for obstructive sleep apnea (OSA). (**A**) ROC curve for distinguishing adult OSA from subjects without OSA. ROC curves for combination of 4-hydroxypentenoic acid, arabinose, glycochenodeoxycholate-3-sulfate, isoleucine, serine, and xanthine. (**B**) ROC curve for distinguishing adult OSA from subjects with simple snorers (SS). ROC curves for the combination of 4-hydroxypentenoic acid, 5-dihydrotestosterone sulfate, serine, spermine, and xanthine.

A total of 28 metabolites changed in patients with OSA compared with those without OSA (Supplementary Table 4, VIP > 1 and *p* < 0.05, respectively). Receiver operating characteristic (ROC) and logistic regression analyses were used to disclose the most qualified metabolic candidates. We generated a model that included six variables (4-hydroxypentenoic acid, arabinose, GCDCA-3-sulfate, isoleucine, serine, and xanthine) as independent predictors of OSA as follows: probability 1 = exp (−0.048 + 0.987 [4-hydroxypentenoic acid] + 0.886 [arabinose] −1.286 [GCDCA-3-sulfate] + 2.131 [isoleucine] −2.071 [serine] −1.106 [xanthine]). Sensitivity and specificity were 75% and 78%, respectively. The area under ROC curve (AUC) and 95% confidence interval (CI) were 0.835 (0.757–0.897) for distinguishing adults with OSA from subjects without OSA (Fig. 3A). Using the same procedure, another five-variable model (AUC = 0.878, 95% CI = 0.792–0.937, probability 3 = exp (6.176 + 1.437 [4-hydroxypentenoic acid [−2.378 [5-dihydrotestosterone sulfate] −2.415 [serine] −0.022 [spermine] −1.703 [xanthine]) distinguished OSA from SS (Fig. 3B). Sensitivity and specificity were 85% and 80%, respectively.

In addition, we combined the aforementioned fitted probabilities with ESS (we also used a forward stepwise logistic regression analysis to generate the new fitted probability) to achieve a more accurate optimum diagnostic cut-off point. However, the results showed that even combined with ESS, the potential false positives and negatives seemed not to be reduced (Supplementary Tables 7 and 8).

**Figure 4:**
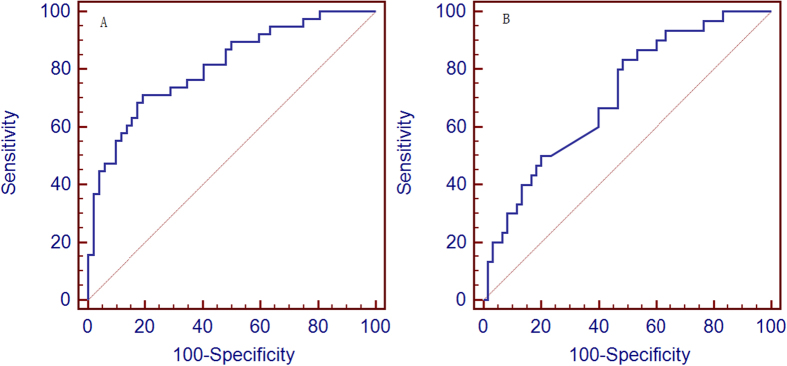
Receiver-operator characteristic (ROC) curve of the composite biomarker panel for moderate to severe obstructive sleep apnea (OSA) and severe OSA. (**A**) ROC curve for distinguishing moderate to severe OSA from mild OSA and SS. ROC curves for the combination of 2-methoxy-4-methylphenol, 3-aminosalicylic acid, 3-hydroxyanthranilic acid and 4-hydroxypentenoic acid. (**B**) ROC curve for distinguishing severe OSA from non-severe OSA. ROC curves for the combination of 3-hydroxyphenylacetic acid and 3-methyl-3-hydroxybutyric acid.

Further, we analyzed 34 metabolites that distinguished the patients with moderate to severe OSA (AHI ≥ 15 events per hour) from those with mild OSA and SS (AHI < 15 events per hour) (Supplementary Table 5). Using the probability = exp(−2.282−1.692 [2-methoxy-4-methylphenol] + 0.415 [3-aminosalicylic acid] + 1.311 [3-hydroxyanthranilic acid] + 0.852 [4-hydroxypentenoic acid]), we were able to distinguish moderate to severe OSA from mild OSA and SS [Sensitivity = 71.1%, Specificity = 80.8%, AUC (CI) = 0.809 (0.713~0.884)] (Fig. 4A). We also identified 29 metabolites that distinguished patients with severe OSA (AHI ≥ 30 events per hour) from non-severe OSA (mild to moderate OSA and SS) (AHI < 30 events per hour) (Supplementary Table 6). Using the probability = exp(−0.532−0.504 [3-hydroxyphenylacetic acid] + 0.116 [3-methyl-3-hydroxybutyric acid]), we could distinguish moderate to severe OSA from mild OSA and SS [Sensitivity = 83.3%, Specificity = 51.7%, AUC (CI) = 0.708 (0.602~0.799)] (Fig. 4B).

## Discussion

Using an integrated MS-based metabolic profiling approach, our study showed that OSA and SS are associated with several altered urinary metabolites. Several potential metabolic pathways were associated with shifting sleep disorders. Importantly, a metabolite panel could potentially be used as a diagnostic tool to distinguish OSA from normal subjects, even SS.

Consistent with former studies, we also found that subjects with OSA had dyslipidemia as shown by higher total cholesterol (TC), triglycerides (TG), low-density lipoprotein cholesterol (LDL-C) and lower high-density lipoprotein cholesterol (HDL-C) levels. In addition, the metabolomics method revealed that several fatty acids and their derivatives were elevated in patients with SS and OSA. Several mechanisms might explain these results. 1) Intermittent hypoxemia (IH) could activate the sympathetic nervous system, and the activated sympathetic nervous system could stimulate adipose tissue lipolysis. During lipolysis, multiple circulating fatty acids are generated. 2) Cells under hypoxia are mainly dependent on anaerobic glycolysis; thus, fatty acids are not fully utilized for energy^16^. 3) IH may impair insulin secretion and adipocyte insulin sensitivity; thus, the anti-lipolytic function of insulin could be compromised^17^,^18^. Fatty acid biosynthesis was also elevated in patients with OSA. A gene expression analysis showed that chronic IH could upregulate multiple genes controlling fatty acid biosynthesis^19^. Surges in nocturnal free fatty acids in patients with OSA may accelerate the progression of underlying heart disease, and supplemental oxygen prevents elevated free fatty acids^20^. Thus, elevated fatty acids and fatty acid biosynthesis may play an important role in the development of cardiovascular and metabolic diseases in patients with OSA.

Glucose metabolic disorder is a typical symptom of patients with OSA and usually occurs with an increase in glucose and insulin resistance. The results of our study were consistent with this notion. Furthermore, the metabolomics study revealed that arabinose, arabitol, cellobiose, glyceraldehyde, and threitol were higher in the SS and OSA groups than in the control group. 2-Butenedioic acid, a TCA cycle intermediate, was significantly lower in the SS and OSA groups than in the control group. Conversely, glycolytic products including lactic acid were higher in the SS and OSA groups than in the control group. When the TCA cycle cannot supply sufficient ATP due to hypoxia, glycolysis compensates by enhancing its activity.

Nucleotides participate in most metabolic reactions as coenzymes. Disturbed nucleic acid metabolism was also observed in patients with OSA. Some rodent and cell studies have demonstrated that IH can activate xanthine oxidase and subsequently increases reactive oxygen species^21^,^22^. Thus, these altered nucleic metabolites could reflect excessive oxidative stress and nuclear and mitochondrial dysfunction. Myo-inositol is an essential component of the plasma membrane that acts as an intracellular second messenger. The myo-inositol level was significantly higher in the OSA and SS groups and could be part of a compensatory response for IH-induced skeletal muscle injury in patients with OSA.

Branch chain amino acids (BCAA, including isoleucine and valine) were higher in the OSA group than in the SS group and healthy controls. Importantly, the accumulation of downstream BCAA metabolites causes mitochondrial dysfunction. Mitochondrial dysfunction is a predictive factor for oxidative stress in patients with OSA^23^. Isoleucine and valine are significantly higher among obese patients and those with diabetes mellitus than normal subjects^24^,^25^. Elevated BCAA levels are also suggested to be a predictor of diabetes mellitus^26^. These findings indicate the importance of mitochondrial function, the interrelationships among our identified metabolites, and their potential roles regulating abnormal metabolic status in OSA.

Under a 6-week IH condition, a higher abundance of *Firmicutes* and lower abundance of *Bacteroidetes* and *Proteobacteria phyla* were found in IH-exposed mice than controls^27^. Sleep fragmentation, another OSA characteristic, can alter the microbial community structure in mice^28^. A causal relationship between gut dysbiosis and OSA-related hypertension has been observed^29^. OSA is associated with increased endotoxemia and impaired gut barrier function in children^30^,^31^. Evidence from rodent and clinical studies shows that OSA is associated with alterations in the gut microbiome, which in turn influence metabolites (i.e., GCDCA-3-sulfate and TMAO) due to the altered gut microbiome. GCDCA-3-sulfate is a bile acid associated with pathological progression of liver dysfunction^32^. TMAO is another gut microbial-dependent metabolite, which is elevated in patients with chronic kidney disease and associated with cardiovascular disease^33^,^34^. Thus, we suggest that altered intestinal flora diversity may be a pivotal link between OSA and the liver and cardiovascular or renal complications.

Full-night PSG is recognized as a standard diagnostic tool. However, it has apparent drawbacks including inconvenience to patients and an onerous workload for technicians. Thus, development of an accurate and noninvasive method is urgently needed. Urine, which is easy and noninvasive to collect, has been widely applied for clinical diagnostic testing. Several proteomic studies reported that certain urine proteins cluster and could be used to screen for pediatric OSA^35^,^36^. Gozal *et al.*^35^ performed a series of proteomics studies and found that using a panel of uromodulin, urocortin-3, orosomucoid-1, and kallikrein yielded sensitivity (specificity) of 95% (100%) for pediatric OSA. In this metabolomics study, we also identified a metabolomics profiling panel consisting of metabolites (4-hydroxypentenoic acid, arabinose, glycochenodeoxycholate-3-sulfate, isoleucine, serine, and xanthine) with moderate power for diagnosing adult OSA. In addition, the combination of 5-dihydrotestosterone sulfate, serine, spermine, and xanthine could be used to distinguish OSA from SS.

Our identification of candidate urinary biomarkers of OSA may enable a simplified and cost-effective diagnostic algorithm that might be feasible for other hospitals/laboratories, and may be cheaper than PSG. In the future, it may be possible to develop a diagnostic biomarker panel to characterize OSA. Using this panel, the OSA-suspected patients will be quickly detected by means of analysis of the morning urine sample. The cost of this type of analysis will be lower than PSG: in China PSG costs half of the average monthly salary.

Despite several interesting findings that explain the differences in metabolites among patients with SS, patients with OSA, and normal subjects, several limitations in this study should be addressed. First, the relatively small sample size in this pilot exploratory study may limit the power to detect small changes in some metabolites. Second, to reduce the effect of potential confounders, the three cohorts (normal group, simple snorers and OSA) in this study were matched for age, sex, and BMI. However, such a stringent design, which was intended to reduce intra-group variations in urinary metabolomics, will require further validation in clinical referral OSA populations for whom no strict exclusion criteria would be implemented. Coexisting diseases that may confound the metabolomics results will also need to be ascertained.

In summary, we successfully used a global metabolomics approach to identify multiple metabolic pathways with differences among patients with SS, patients with OSA, and healthy controls. Our results suggest that a panel of urinary metabolite markers could be used as a moderate diagnostic tool for OSA. Further prospective community studies with larger sample sizes are warranted to verify our findings.

## Materials and Methods

### Study population

This study was conducted in accordance with the Declaration of Helsinki and approved by the Ethics Committee of Shanghai Jiao Tong University Affiliated Sixth People’s Hospital. Written informed consent was obtained from each participant. We established the BioBank of OSA in 2007 and have greater than 6,000 individual samples. We have collected blood and urine samples from all patients who underwent sleep monitoring in our sleep center. We call this large cohort the “Shanghai Sleep Health Study” (SSHS)^37^. The SSHS is a dynamic and ongoing study. The normal subjects were recruited from Xuhui District, Shanghai, China and all of them had standard PSG. None of the normal subjects had a history of disturbed sleep disorders or self-reported/witnessed snoring. The samples of OSA and simple snorers, well-matched for age, BMI and sex, used in this metabolomics study were chosen from among the SSHS cohort. Exclusion criteria were: 1) systemic disease (i.e., renal, liver, cardiovascular, pulmonary, or neurological disease) and 2) treatment for SS or OSA (i.e., surgery, continuous positive airway pressure, or an oral appliance). Participants who took hormone replacement therapy or any other therapy that could influence the metabolic results or measurements of metabolites were also excluded. In addition, all participants were nonsmokers, had no alcohol problems, and had no medical history of hypertension, diabetes, or dyslipidemia. Anthropometric parameters including height, weight, neck circumference, waist circumference, and hip circumference were measured; BMI was calculated as weight/height^2^ (kg/m^2^). Blood pressure was measured in a quiet room three times at 5-min intervals. Systolic blood pressure and diastolic blood pressure were recorded as the mean of three measurements.

### Excessive daytime sleepiness evaluation and full-night polysomnography

Each participant completed a Chinese version of the eight-item Epworth Sleepiness Scale questionnaire prior to the sleep study^38^. In-laboratory PSG (Alice 5; Respironics, Pittsburgh, PA, USA) was used to assess nocturnal sleep. This sleep system utilizes electroencephalography, left and right electrooculography, submental electromyography, nasal and oral airflow, snoring sounds, electrocardiography, thoracic/abdominal movements, pulse oxygen saturation, and body position to measure various parameters. These respiratory parameters were recorded automatically and continuously from 10 PM to 6 AM. The PSG indices included AHI, oxygen desaturation index, mean oxygen saturation, lowest pulse oxygen saturation, and arousal index. Apnea was defined as cessation in airflow of at least 90% of baseline for ≥10 s, and hypopnea was defined as a drop in airflow of >30% with oxygen desaturation of ≥4%. The AHI was calculated using the number of respiratory events per hour of sleep according to the American Academic Sleep Medicine criteria. All PSG indices were manually checked by a technician. After the sleep study, the subjects were divided into three groups: 1) patients with OSA: the above-mentioned patients recruited from the hospital had an AHI of ≥5 (n = 60), 2) patients with SS: the above-mentioned patients with an AHI of <5 (n = 30), and 3) normal subjects: the aforementioned preliminary normal subjects with an AHI of <5 (n = 30). The age, BMI, and sex distribution of the SS and normal groups were comparable with those in the OSA group.

### Urine and blood collection

Samples were taken from all patients who underwent a sleep study. Fasting blood and urine samples were collected. First, midstream urine specimens were collected from all participants at the same time of day (7:00 AM). The urine samples (1 mL) were immediately stored in tubes at −80 °C until UPLC-Q-TOF-MS and GC-TOF-MS analyses. A fasting blood sample was also collected after PSG measurement (7:00 AM) from each participant, and the sample was sent immediately for clinical biochemical analysis.

### Biochemical measurements

A lipid profile [i.e., TC, TG, HDL-C, LDL-C, apolipoprotein (apo) A-I, apoB, apoE, and lipoprotein (a)] and serum glucose were measured in the hospital clinical laboratory using an H-7600 autoanalyzer (Hitachi, Tokyo, Japan). Fasting serum insulin was measured using an immunoradiological method. Homeostasis model assessment of insulin resistance (HOMA-IR) was calculated by the following formula: HOMA-IR = fasting serum insulin (μU/mL) × fasting plasma glucose (mmol/L)/22.5^39^.

### Urinary sample preparation

The urinary metabolites were extracted as described previously with minor modifications^40^. For the UPLC-Q-TOF-MS analysis, a 400 μL aliquot of methanol and acetonitrile (1:9, v/v) and 20 μL of internal standard (30 μg/mL p-chlorophenylalanine in water) were added to 200 μL of urine. This mixture was vortexed for 2 min and centrifuged at 12,000 × *g* for 15 min. The supernatant was transferred to an LC sampling vial for analysis. For the GC-TOF-MS analysis, 10 μL of urease (30 U/10 μL, type C) was added to a 100 μL urine sample and kept at 37 °C for 15 min to catalyze urea. Next, 300 μL of methanol was added remove the remaining urease. A 10 μL aliquot of internal standard (100 μg/mL p-chlorophenylalanine in water) was added, followed by centrifugation at 12,000 × *g* for 5 min. A 200 μL aliquot of supernatant was transferred to a GC sampling vial for trimethyl-silyation derivatization prior to analysis.

### Instrumental analysis and metabolite identification

Metabolomics profiling with UPLC-Q-TOF-MS and GC-TOF-MS was performed following our previous publication and optimized^40^. The urine samples were run in random order, and 1 quality control and 1 blank sample were run after each 10 urine samples. A Waters ACQUITY UPLC system equipped with a binary solvent delivery manager and a sample manager (Waters Corp., Milford, MA, USA) was coupled to a Q-TOF mass spectrometer equipped with an electrospray interface (Waters) as the LC-MS platform. An Agilent 6890N gas chromatography coupled with a Pegasus HT TOF MS (Leco Corp., St. Joseph, MI, USA) was used as the GC-TOF-MS platform.

The MS data were analyzed to identify potential discriminate variables. The UPLC-Q-TOF-MS raw data were analyzed using MarkerLynx applications manager ver. 4.1 (Waters, Manchester, UK), and the MS files from the GC-TOF-MS analysis were analyzed using ChromaTOF ver. 3.30 software (Leco). The resulting three-dimensional data set included sample information, peak retention time, and peak intensities.

The compounds were annotated using our in-house library containing more than 800 mammalian metabolite standards. Metabolites were detected in the UPLC-Q-TOF-MS data by comparison with the accurate mass and the retention time of reference standards in our in-house library and the accurate mass of compounds obtained from web-based resources, such as the Human Metabolome Database (http://www.hmdb.ca/). Metabolites were detected in the GC-TOF-MS data by comparison with the mass fragments and retention time of reference standards in our in-house library or mass fragments in the NIST 05 Standard mass spectral databases in NIST MS search 2.0 (NIST, Gaithersburg, MD, USA) software with similarity of >70%.

### Statistical analysis

The statistical analysis was performed using SPSS ver. 20.0 (IBM Corp., Armonk, NY, USA), Medcalc ver. 12.7.3 (Medcalc, Mariakerke, Belgium), or SIMCA-P 13.0 (Umetrics, Umea, Sweden) software packages after normalizing the data to total intensity. Principal component analysis and OPLS-DA were performed to visualize the metabolic differences between the normal, the SS and the OSA groups. Significantly altered metabolites with a VIP of >1 in the OPLS-DA models and Student’s *t*-test (*p* < 0.05) were selected. The fold change was the intensity mean value ratio of the two groups. A forward stepwise logistic regression analysis was performed to select the strongest variables (metabolites), and the fitted probability was generated. A ROC curve analysis was then used to determine the cut-off values that provided maximum diagnostic efficiency. A *p*-value of <0.05 indicated statistical significance.

## Additional Information

**How to cite this article**: Xu, H. *et al.* Metabolomics Profiling for Obstructive Sleep Apnea and Simple Snorers. *Sci. Rep.* **6**, 30958; doi: 10.1038/srep30958 (2016).

## Supplementary Material

Supplementary Information

## Figures and Tables

**Table 1:** Demographic characteristics of the enrolled subjects.

Index	Normal group (n = 30)	SS group (n = 30)	OSA group (n = 60)	p_1_	p_2_	p_3_
Age (years)	44.90 ± 9.48	41.53 ± 12.20	42.73 ± 13.13			
Male sex, n	15	15	30			
BMI (Kg/m^2^)	23.08 ± 2.25	22.97 ± 2.27	23.71 ± 2.26			
TC (mmol/L)	4.23 ± 1.01	4.45 ± 0.81	4.72 ± 0.76		*	
TG (mmol/L)	1.19 ± 1.19	1.37 ± 0.81	2.06 ± 2.06		*	
HDL (mmol/L)	1.16 ± 0.26	1.14 ± 0.22	1.08 ± 0.23			
LDL (mmol/L)	2.50 ± 0.69	2.84 ± 0.68	2.88 ± 0.63		*	
ApoA-I (g/L)	1.19 ± 0.18	1.27 ± 0.21	1.30 ± 0.22		*	
ApoB (g/L)	0.68 ± 0.12	0.76 ± 0.16	0.84 ± 0.16	*	‡	*
ApoE (mg/dL)	4.02 ± 1.56	3.87 ± 0.98	4.95 ± 1.85		*	†
Lpa (mg/L)	10.84 ± 12.65	16.62 ± 24.10	17.48 ± 18.59			
Glucose (mmol/L)	4.96 ± 0.51	5.05 ± 0.33	5.10 ± 0.72			
Insulin (μU/mL)	6.52 ± 3.05	8.49 ± 3.93	11.39 ± 7.49	*	‡	
HOMA-IR	1.45 ± 0.71	1.91 ± 0.90	2.61 ± 1.70	‡	‡	
SBP (mmHg)	119.43 ± 14.56	118.50 ± 9.58	124.87 ± 11.45			*
DBP (mmHg)	75.43 ± 8.56	73.83 ± 8.65	78.52 ± 9.71			*
NC (cm)	35.08 ± 3.58	36.10 ± 3.47	37.31 ± 3.28		†	
WC (cm)	81.15 ± 16.30	84.33 ± 7.07	89.31 ± 9.03		†	†
HC (cm)	94.80 ± 5.77	96.15 ± 5.19	96.03 ± 12.84			
ESS	2.30 ± 2.51	6.77 ± 6.00	7.97 ± 5.64	‡	‡	
AHI	0.62 ± 0.60	1.98 ± 1.35	34.40 ± 24.33	‡	‡	‡
Mean SaO_2_	96.88 ± 1.60	95.90 ± 1.52	94.90 ± 1.95	*	‡	*
LSpO_2_	94.47 ± 3.69	93.40 ± 3.58	82.33 ± 8.23		‡	‡
ODI	0.89 ± 0.99	2.56 ± 1.84	34.54 ± 25.59	‡	‡	‡
ArI	10.90 ± 4.48	16.23 ± 9.68	25.49 ± 15.63	†	‡	†

Abbreviations: SS, simple snorers; OSA, obstructive sleep apnea; BMI, body mass index; TC, total cholesterol; TG, triglyceride; HDL-C, high-density lipoprotein cholesterol; LDL-C, low-density lipoprotein cholesterol; apoA-I, apolipoprotein A-I; apoB, apolipoprotein B; apoE, apolipoprotein E; Lp(a), lipoprotein (a); HOMA-IR, homeostasis model assessment of insulin resistance; SBP, systolic blood pressure; DBP, diastolic blood pressure; NC, neck circumference; WC, waist circumference, HC, hip circumference; ESS, Epworth sleepiness score; AHI, apnea–hypopnea index; SaO_2_, oxygen saturation; LSpO_2_, lowest pulse oxygen saturation; ODI, oxygen desaturation index; ArI, arousal index.

Note: p_1_: Normal group vs. SS group; p_2_: Normal group vs. OSA group; p_3_: SS vs. OSA group. *p < 0.05, ^†^p < 0.01, ^‡^p < 0.001.
