# Supplementary material for: Metabolomics Profiling for Obstructive Sleep Apnea and Simple Snorers
Source: Sci Rep. 2016 Aug 2;6:30958. doi: 10.1038/srep30958 (PMC4969608; doi:10.1038/srep30958)
Supplement: Supplementary Information [file srep30958-s1.pdf]

Metabolomics Profiling for Obstructive Sleep Apnea and Simple Snorers

Huajun Xu, MD [1,2,3*], Xiaojiao Zheng, PhD [4*], Yingjun Qian, MD [1,2,3*], Jian Guan MD, PhD [1,2,3], Hongliang Yi MD, PhD [1,2,3], Jianyin Zou MD, PhD [1,2,3], Yuyu Wang, MD, [1,2,3], Lili Meng, MD[1,2], Aihua Zhao, PhD [4], Shankai Yin, MD, PhD[1,2,3], Wei Jia, PhD [3,4]

1 Department of Otolaryngology Head and Neck Surgery & Center of Sleep Medicine, Shanghai Jiao Tong University Affiliated Sixth People's Hospital, Yishan Road 600, 200233 Shanghai, China.

2 Otolaryngological Institute of Shanghai Jiao Tong University, Yishan Road 600, 200233 Shanghai, China.

3 Clinical Research Center, Shanghai Jiao Tong University School of Medicine, South Chongqing Road 225, 200020 Shanghai, China.

4 Center for Translational Medicine, Shanghai Jiao Tong University Affiliated Sixth People's Hospital, Yishan Road 600, 200233 Shanghai, China.

*these authors contributed equally to this paper.

Corresponding author: Shankai Yin, MD, PhD (Email: skyin@sjtu.edu.cn) and Wei Jia, PhD (Email: wjia4226@hotmail.com)

1) Introduction to Metabolomics:

Metabolomics, defined as the measurement of multiparametric metabolic responses of a biological compartment or a living system to pathophysiological stimuli or genetic modification, is an effective tool for toxicological studies, the potential biomarker identification of diseases, and the identification of regulatory network pathways in plants and animals. Selectively monitoring characteristic variation of endogenous metabolites (molecular weight < 1,000 Da) in a single cell, biofluids, or tissue, coupled with widely ascribed multivariate statistical analysis techniques, enables such a state-of-the-art tool for probing the network integrity, systems diversity, and complexity of human individuals.

2) Introduction to Metabolomics analysis:

Multivariate analyses, unsupervised principal component analysis (PCA) and supervised orthogonal partial least-squares discriminant analysis (OPLS-DA), were performed in this study. The discrimination of samples could be visualized in the score plots. PCA is a conventional unsupervised multivariate analysis method aiming for dimension reduction. This technique transforms an original set of correlated variables to a new set of uncorrelated variables, called principal components (PCs). This transformation is defined in such a way that the first principal component has the largest possible variance (that is, accounts for as much of the variability in the data as possible), and each succeeding component in turn has the highest variance possible under the constraint that it is orthogonal to the preceding components. The scores of PCs are coordinates of observations (samples in this report) when it is projected on the projection plane or hyper plane of a model.

3) Detailed information explaining Figure 1:

The OPLS-DA model was generated with one predictive component, and one orthogonal component, to discriminate different groups. R2X and R2Y represent the fraction of the variance of the X variables (all identified metabolites in this report) and Y variables (control subjects, SS, and OSA in this report) explained by the model, while Q2Y suggests the predictive performance of the model.

Supplementary Table 1. Metabolites Identified in the Data Set that Distinguished Subjects with Simple Snorers from Normal Subjects

Pathway	Metabolite	VIP[a]	FC[b]	*p*-value[c]
Fatty acid metabolism	6-keto-decanoylcarnitine	1.87	0.77	2.78×10^{-2}
	8-hydroxy-5,6-octadienoic acid	1.94	1.44	2.22×10^{-2}
	heptanedioic acid	1.98	4.14	1.97×10^{-2}
	menthyl acetoacetate	2.48	1.80	3.04×10^{-3}
	perillyl acetate	2.52	1.67	2.48×10^{-3}
Phospholipid biosynthesis	cytidine 5'-diphosphocholine	1.77	1.70	3.82×10^{-2}
	myo-inositol	1.70	1.36	4.67×10^{-2}
Amino acid metabolism	3-hydroxyanthranilic acid	1.91	0.77	2.42×10^{-2}
	5-hydroxytryptophan	2.05	0.84	1.53×10^{-2}
	aspartyl-serine	2.68	1.49	1.26×10^{-3}
	Ile-Thr	2.00	5.53	1.80×10^{-2}
	Methionine	1.74	1.32	4.17×10^{-2}
Carbohydrate metabolism	Arabitol	2.06	1.33	1.47×10^{-2}
	Xylitol	2.55	0.72	2.22×10^{-3}
Tricarboxylic acid cycle	2-butenedioic acid	2.25	0.21	7.56×10^{-3}
Indoles and derivatives	3-indoleacetonitrile	1.71	1.39	4.53×10^{-2}
	Indole	2.21	0.76	8.61×10^{-3}
Glutamate metabolism	4-hydroxybutyric acid	2.58	1.64	1.94×10^{-3}
Steroidogenesis	5-dihydrotestosterone sulfate	2.07	1.35	1.42×10^{-2}
Nucleic acid metabolism	Hypoxanthine	2.45	0.64	3.39×10^{-3}
	1,3-dimethyluracil	1.71	1.46	4.48×10^{-2}

Note: [a]Variable importance in projection (VIP) was calculated by orthogonal partial least-squares discriminant analysis with a threshold of 1.0. [b]Fold change (FC) was obtained from the average value of the simple snorers (SS) group compared with that of the normal group. An FC of > 1 indicates a higher level of the urinary metabolite in the SS group than in controls, while an FC of < 1 indicates a lower level in the SS group. [c]*p*-values were calculated using Student's *t*-test.

Supplementary Table 2. Metabolites Identified in the Data Set that Distinguished Patients with Obstructive Sleep Apnea from Normal Subjects Identified in the Data

Set

Pathway	Metabolite	VIP[a]	FC[b]	p-value[c]
Fatty acid metabolism	2,4-dihydroxybutyric acid	1.94	1.25	9.70×10^{-3}
	2-hydroxy-3-methylbutyric acid	1.63	1.35	3.09×10^{-2}
	3,4-dihydrxoybutyric acid	1.99	1.27	7.87×10^{-3}
	6-aminocaproic acid	1.49	1.39	4.94×10^{-2}
	pentanoic acid	1.73	1.63	2.17×10^{-2}
	glyceraldehyde	1.52	1.25	4.50×10^{-2}
Phospholipid biosynthesis	cytidine 5'-diphosphocholine	1.73	2.14	2.14×10^{-2}
	ethanolamine	2.03	0.85	6.70×10^{-3}
	myo-inositol	2.35	1.53	1.56×10^{-3}
Carbohydrate metabolism	2,3-dihydrxoypropanoic acid	1.69	1.39	2.47×10^{-2}
	arabinose	1.53	1.28	4.34×10^{-2}
	arabitol	2.38	1.50	1.37×10^{-3}
	cellobiose	1.61	1.41	3.29×10^{-2}
	threitol	1.78	1.19	1.79×10^{-2}
Amino acid metabolism	hydroxyprolyl-methionine	1.50	1.26	4.69×10^{-2}
	methylcysteine	1.56	0.37	3.88×10^{-2}
	threoninyl-methionine	1.51	1.20	4.54×10^{-2}
	serine	1.53	0.88	4.25×10^{-2}
	valine	2.29	1.18	2.11×10^{-3}
	isoleucine	2.60	1.30	4.39×10^{-4}
	trimethylamine-N-oxide	1.48	2.40	4.99×10^{-2}
Tricarboxylic acid cycle	2-butenedioic acid	1.53	0.48	4.35×10^{-2}
Bile acid metabolism	glycochenodeoxycholate-3-sulfate	2.85	0.63	1.04×10^{-4}
Glutamate metabolism	4-hydroxybutyric acid	2.13	1.96	4.48×10^{-3}
Nucleic acid metabolism	hypoxanthine	2.53	0.62	6.17×10^{-4}
	xanthine	1.56	0.81	3.90×10^{-2}
	uridine	1.67	5.25	2.71×10^{-2}
Indoles and derivatives	indole-3-acetamide	1.73	0.86	2.20×10^{-2}
Spermine biosynthesis	putrescine	1.53	3.01	4.33×10^{-2}
	spermine	1.63	0.36	3.12×10^{-2}

Note: [a]Variable importance in projection (VIP) was calculated by orthogonal partial least-squares discriminant analysis with a threshold of 1.0. [b]Fold-change (FC) was obtained from the average value of the obstructive sleep apnea (OSA) group

compared with that of the normal group. An FC of > 1 indicates a higher level of the urinary metabolite in the OSA group than in controls, while an FC of < 1 indicates a lower level in the OSA group. [c]*p*-values were calculated from Student's *t*-test.

Supplementary Table 3. Metabolites Identified in the Data Set that Distinguished Patients with Obstructive Sleep Apnea (OSA) from Those with Simple Snoring (SS)

Pathway	Metabolite	VIP[a]	FC[b]	*p*-value[c]
Fatty acid metabolism	3-hydroxybutyric acid	1.99	1.34	2.31×10^{-2}
	3-methyl-3-hydroxybutyric acid	1.86	2.36	3.37×10^{-2}
	4-hydroxypentenoic acid	1.97	1.40	2.47×10^{-2}
	N-nonanoylglycine	1.83	0.48	3.71×10^{-2}
Amino acid metabolism	aspartyl-serine	2.18	0.76	1.26×10^{-2}
	methionine	2.31	0.76	7.94×10^{-3}
	serine	2.10	0.84	1.63×10^{-2}
	threoninyl-methionine	1.87	1.20	3.27×10^{-2}
Tricarboxylic acid cycle	lactic acid	1.76	1.59	4.46×10^{-2}
	2-methyl-2-hydroxypropanoic acid	1.85	1.22	3.51×10^{-2}
Bile acid metabolism	glycochenodeoxycholate-3-sulfate	1.80	0.78	4.07×10^{-2}
Steroidogenesis	5-dihydrotestosterone sulfate	2.44	0.74	5.00×10^{-3}
Spermine biosynthesis	spermine	1.91	0.04	2.92×10^{-2}
Tryptophan metabolism	5-hydroxyindoleacetic acid	1.95	1.19	2.63×10^{-2}
Porphyrin metabolism	urobilinogen	2.20	0.16	1.18×10^{-2}
	5-aminolevulinic acid	1.96	1.26	2.55×10^{-2}
Nucleic acid metabolism	Inosine	1.98	0.31	2.39×10^{-2}
	xanthine	2.07	0.75	1.81×10^{-2}

Note: [a]Variable importance in projection (VIP) was calculated by orthogonal partial least-squares discriminant analysis with a threshold of 1.0. [b]Fold-change (FC) was obtained from the average value of the OSA group compared with that of the SS group. An FC of > 1 indicates a higher level of the urinary metabolite in the OSA group than in the SS group, while an FC value of < 1 indicates a lower level in the OSA group. [c]*p*-values were calculated from Student's *t*-test.

Supplementary Table 4. Metabolites Identified in the Data Set that Distinguished Patients With and Without Obstructive Sleep Apnea (OSA)

Pathway	Metabolite	VIP[a]	FC[b]	*p*-value[c]
Fatty acid metabolism	2-hydroxy-3-methylbutyric acid	1.69	1.27	2.86×10^{-2}
	3,4-dihydrxoybutyric acid	1.99	1.19	9.67×10^{-3}
	3-hydroxybutyric acid	1.93	1.29	1.20×10^{-2}
	4-hydroxypentenoic acid	1.79	1.28	1.99×10^{-2}
Phospholipid biosynthesis	cytidine 5'-diphosphocholine	1.62	1.58	3.53×10^{-2}
	ethanolamine	1.63	0.90	3.47×10^{-2}
	myo-inositol	1.92	1.30	1.26×10^{-2}
Carbohydrate metabolism	2,3-dihydrxoypropanoic acid	2.10	1.34	6.18×10^{-3}
	arabinose	1.54	1.19	4.63×10^{-2}
	arabitol	2.06	1.29	7.22×10^{-3}
	cellobiose	1.91	1.34	1.28×10^{-2}
	maltose	1.64	1.36	3.35×10^{-2}
	threitol	1.90	1.15	1.35×10^{-2}
Amino acid metabolism	alanine	1.78	1.23	2.04×10^{-2}
	isoleucine	2.18	1.21	4.34×10^{-3}
	serine	2.03	0.86	8.18×10^{-3}
	threoninyl-methionine	2.03	1.20	8.16×10^{-3}
	trimethylamine N-oxide	1.78	1.98	2.11×10^{-2}
	valine	2.06	1.12	7.08×10^{-3}
Tryptophan metabolism	5-hydroxyindoleacetic acid	1.65	1.15	3.27×10^{-2}
Tricarboxylic acid cycle	lactic acid	1.87	1.58	1.51×10^{-2}
Bile acid metabolism	glycochenodeoxycholate-3-sulfate	2.51	0.70	9.72×10^{-4}
Spermine biosynthesis	putrescine	1.91	2.52	1.30×10^{-2}
Glutamate metabolism	4-hydroxybutyric acid	1.95	1.48	1.12×10^{-2}
Tyrosine metabolism	vanillic acid	2.27	1.30	2.93×10^{-3}
Nucleic acid metabolism	hypoxanthine	1.83	0.75	1.74×10^{-2}
	inosine	1.71	0.34	2.61×10^{-2}
	xanthine	2.02	0.78	8.42×10^{-3}

Note: [a]Variable importance in projection (VIP) was calculated by orthogonal partial

least-squares discriminant analysis with a threshold of 1.0. [b]Fold-change (FC) was obtained from the average value of the OSA group compared with that of the non-OSA group. An FC of > 1 indicates a higher level of the urinary metabolite in the OSA group than in the non-OSA group, while an FC of < 1 indicates a lower level in the OSA group. [c]*p*-values were calculated from Student's *t*-test.

Supplementary Table 5. Metabolites Identified in the Data Set that Distinguished Patients with Moderate to Severe Obstructive Sleep Apnea (OSA) from those with Mild OSA and Simple Snorers (SS)

Pathway	Metabolite	VIP[a]	FC[b]	*p*-value[c]
Fatty acid metabolism	4-hydroxypentenoic acid	2.25	1.28	3.43×10^{-3}
	5-hydroxyvaleric acid	1.92	1.27	1.32×10^{-2}
	6-aminocaproic acid	1.94	0.74	1.22×10^{-2}
	glyceraldehyde	1.65	0.46	3.41×10^{-2}
	glycerol	1.84	0.37	1.80×10^{-2}
	itaconic acid	2.28	1.75	3.02×10^{-3}
	stearic acid	1.95	1.28	1.19×10^{-2}
Phospholipid biosynthesis	myo-inositol	1.77	0.52	2.26×10^{-2}
Amino acid metabolism	2-phenylacetamide	1.55	0.78	4.67×10^{-2}
	3-aminosalicylic acid	1.58	0.80	4.22×10^{-2}
	guanidine acetic acid	2.04	0.46	8.23×10^{-3}
	Isoleucine	1.94	0.59	1.21×10^{-2}
	N-acetylserine	1.86	0.84	1.66×10^{-2}
	N-gamma-glutamyl-methionine	1.63	0.88	3.70×10^{-2}
Tyrosine metabolism	2,5-dihydroxybenzoic acid	2.13	1.37	5.88×10^{-3}
	tyrosine	1.99	1.25	1.03×10^{-2}
	vanillic acid	1.61	0.71	3.86×10^{-2}
Tryptophan metabolism	3-hydroxyanthranilic acid	1.60	0.79	4.07×10^{-2}
	tryptophan	2.20	1.33	4.38×10^{-3}
Glutamate metabolism	4-hydroxybutyric acid	1.96	1.51	1.16×10^{-2}
Carbohydrate metabolism	arabinose	1.60	1.34	4.06×10^{-2}
	maltitol	1.70	1.21	2.92×10^{-2}
	ribose	1.77	0.75	2.28×10^{-2}
	xylitol	2.07	1.40	7.39×10^{-3}
Tricarboxylic acid cycle	trans-aconitic acid	2.24	1.31	3.64×10^{-3}

Norepinephrine metabolism	3,4-dihydroxyphenylglycol	1.54	8.58	4.91×10^{-2}
Porphyrin metabolism	5-aminolevulinic acid	1.65	0.47	3.46×10^{-2}
Nucleic acid metabolism	8-hydroxy-7-methylguanine	1.58	1.24	4.27×10^{-2}
	orotidine	2.75	2.30	2.93×10^{-4}
Organic compound	2,5-undecadienal	1.71	1.19	2.84×10^{-2}
	2-aminobenzoic acid	1.66	0.66	3.36×10^{-2}
	2-methoxy-4-methylphenol	1.78	1.24	2.19×10^{-2}
	lanthionine	2.10	0.37	6.55×10^{-3}
	nicotinate D-ribonucleoside	1.87	4.67	1.58×10^{-2}

Note: [a]Variable importance in projection (VIP) was calculated by orthogonal partial least-squares discriminant analysis with a threshold of 1.0. [b]Fold change (FC) was obtained from the average value of the moderate to severe obstructive sleep apnea (OSA) group compared with that of mild OSA and simple snorers (SS). An FC of > 1 indicates a higher level of the urinary metabolite in the moderate to severe OSA group than in the mild OSA and SS groups, while an FC of < 1 indicates a lower level in the mild OSA and SS group. [c]*p*-values were calculated using Student's *t*-test.

Supplementary Table 6. Metabolites Identified in the Data Set that Distinguished Patients with Severe Obstructive Sleep Apnea (OSA) from non-severe OSA.

Pathway	Metabolite	VIP[a]	FC[b]	*p*-value[c]
Fatty acid metabolism	3-methyl-3-hydroxybutyric acid	1.64	0.76	4.97×10^{-2}
	4-hydroxypentenoic acid	2.04	1.24	1.42×10^{-2}
	6-aminocaproic acid	1.82	0.76	2.91×10^{-2}
	glyceraldehyde	1.81	0.40	3.00×10^{-2}
	glycerol	1.74	0.37	3.68×10^{-2}
	stearic acid	2.59	1.36	1.64×10^{-3}
Phospholipid biosynthesis	myo-inositol	1.87	0.48	2.50×10^{-2}
Carbohydrate metabolism	lactose	1.86	1.56	2.56×10^{-2}
	ribose	1.81	0.75	3.01×10^{-2}
	xylitol	2.46	1.46	2.79×10^{-3}
Amino acid metabolism	2-phenylacetamide	1.81	0.75	3.00×10^{-2}
	2-phenylglycine	1.68	1.19	4.40×10^{-2}

	3-aminosalicylic acid	1.90	0.76	2.26×10^{-2}
	glutamine	1.64	1.69	4.98×10^{-2}
	guanidine acetic acid	1.76	0.51	3.52×10^{-2}
	Isoleucine	1.86	0.60	2.59×10^{-2}
	N-acetylserine	1.79	0.85	3.17×10^{-2}
Tyrosine metabolism	2,5-dihydroxybenzoic acid	1.99	1.32	1.68×10^{-2}
	3-hydroxyphenylacetic acid	1.65	1.22	4.85×10^{-2}
	tyrosine	2.48	1.31	2.60×10^{-3}
Glutamate metabolism	4-hydroxybutyric acid	2.54	1.64	1.97×10^{-3}
Microbial metabolism	3-dehydroxycarnitine	1.91	0.66	2.18×10^{-2}
Tryptophan metabolism.	tryptophan	1.83	1.25	2.77×10^{-2}
Tricarboxylic acid cycle	lactic acid	1.83	1.32	2.76×10^{-2}
	trans-aconitic acid	1.77	1.23	3.39×10^{-2}
Nucleic acid metabolism	orotidine	1.92	1.71	2.11×10^{-2}
Norepinephrine metabolism	3,4-dihydroxyphenylglycol	1.98	10.51	1.70×10^{-2}
Organic compound	2-aminobenzoic acid	1.67	0.65	4.53×10^{-2}
	2-methoxy-4-methylphenol	2.00	1.27	1.61×10^{-2}

Note: [a]Variable importance in projection (VIP) was calculated by orthogonal partial least-squares discriminant analysis with a threshold of 1.0. [b]Fold change (FC) was obtained from the average value of the severe obstructive sleep apnea (OSA) group compared with that of the non-severe OSA group. An FC of > 1 indicates a higher level of the urinary metabolite in the severe OSA group than non-severe OSA group, while an FC of < 1 indicates a lower level in the non-severe OSA group. [c]*p*-values were calculated using Student's *t*-test.

Supplementary Table 7. The efficiency of ESS, probability 1 and probability 2 versus PSG for detecting OSA with non-OSA

Criterion	AUC	Sensitivity (%)	Specificity (%)	+LR	-LR	+PV (%)	-PV (%)
ESS	0.679 (0.607~0.778)	58.3 (44.9~70.9)	75.0 (62.1~85.3)	2.33 (1.8~3.0)	0.56 (0.3~0.9)	70 (55.4~82.1)	64.3 (51.8~75.5)
Probability 1	0.835 (0.757~0.897)	75.0 (62.1~85.3)	78.3 (65.8~87.9)	3.46 (2.8~4.2)	0.32 (0.2~0.6)	77.6 (64.7~87.5)	75.8 (63.3~85.8)
Probability 2	0.852 (0.776~0.910)	66.7 (53.3~78.3)	90.0 (79.5~96.2)	6.67 (5.5~8.1)	0.37 (0.2~0.9)	87.0 (73.6~95.1)	73.0 (61.4~82.6)

Abbreviations: PSG, polysomnography; OSA, obstructive sleep apnea; ESS, Epworth Sleepiness Scale; AUC, area under the ROC curve; +LR, positive likelihood ratio; −LR, negative likelihood ratio; +PV, positive predictive value; −PV, negative predictive value.

Note: Probability2 generated from Probability1 with ESS using a forward stepwise logistic regression analysis.

Supplementary Table 8. The efficiency of ESS, probability3 and probability4 versus PSG for detecting OSA with SS

Criterion	AUC	Sensitivity (%)	Specificity (%)	+LR	-LR	+PV (%)	-PV (%)
ESS	0.573 (0.464~0.677)	75.0 (62.1~85.3)	43.3 (25.5~62.6)	1.32 (0.9~2.0)	0.58 (0.3~1.0)	72.6 (59.8~83.1)	46.4 (27.5~66.1)
Probability 3	0.878 (0.792~0.937)	85.0 (73.4~92.9)	80.0 (61.4~92.3)	4.25 (3.5~5.2)	0.19 (0.07~0.5)	89.5 (78.5~96.0)	72.7 (54.5~86.7)
Probability 4	0.878 (0.792~0.937)	85.0 (73.4~92.9)	80.0 (61.4~92.3)	4.25 (3.5~5.2)	0.19 (0.07~0.5)	89.5 (78.5~96.0)	72.7 (54.5~86.7)

Abbreviations: PSG, polysomnography; OSA, obstructive sleep apnea; ESS, Epworth Sleepiness Scale; AUC, area under the ROC curve; +LR, positive likelihood ratio; −LR, negative likelihood ratio; +PV, positive predictive value; −PV, negative predictive value.

Note: Values in parentheses are 95% confidence intervals. Probability 4 generated from Probability 3 with ESS using a forward stepwise logistic regression analysis.
